# Cross-Sectional Cladding Segmentation of Stainless-Steel/Carbon-Steel Clad Wire Rods Using an Improved U-Net with Multi-Scale Attention

**DOI:** 10.3390/ma19112359

**Published:** 2026-06-02

**Authors:** Lei Zeng, Zecheng Zhuang, Geng Zhou, Weiping Lu, Xuehai Qian, Zhen Li, Zhe Gou, Yue Yu, Jianping Tan

**Affiliations:** 1 School of Mechanical and Electrical Engineering, Central South University, Changsha 410083, China; 13698079602@163.com (L.Z.);; 2 State Key Laboratory of Precision Manufacturing for Extreme Service Performance, Central South University, Changsha 410083, China; 3 School of Mechanical and Electrical Engineering, Loudi Vocational and Technical College, Loudi 417000, China; 4 Technology Centre, Guangxi Liuzhou Iron and Steel Group Ltd., Liuzhou 545002, China; 5 Changsha WuJiang Intelligent Innovation Technology Co., Ltd., Changsha 410023, China

**Keywords:** stainless-steel/carbon-steel clad wire rod, cladding segmentation, improved U-Net, attention mechanism, multi-scale feature fusion, bridge cable quality inspection

## Abstract

Accurate cladding segmentation is essential for quantitative quality assessment of stainless-steel/carbon-steel clad wire rods used in bridge cables, yet remains challenging because of weak core–cladding contrast, narrow interfacial transition zones, local cladding-thickness fluctuations, and limited repeatability of manual inspection. This study proposes an improved U-Net framework that integrates residual feature extraction, multi-scale contextual perception, and attention-guided feature refinement for robust cladding identification. A cross-sectional image dataset comprising 18,566 samples was constructed through standardized specimen preparation, chemical color development, image acquisition, pixel-level annotation, and data augmentation. In the proposed model, the original U-Net encoder is replaced with ResNet50 to enhance deep semantic representation, while atrous spatial pyramid pooling and a convolutional block attention module are embedded into the feature-fusion stage to improve boundary discrimination and thin-cladding recognition. On the test set, the model achieved a mean pixel accuracy of 97.29%, cladding intersection over union of 88.82%, and mean intersection over union of 93.72%, outperforming the baseline U-Net by 1.38, 9.19, and 5.17 percentage points, respectively. Ablation and comparative experiments further demonstrate improved boundary continuity, local-detail preservation, and segmentation stability compared with representative CNN-based segmentation models. These findings suggest that the proposed framework provides a practical and reliable vision-based approach for cladding-thickness measurement, eccentricity evaluation, uniformity assessment, and batch quality inspection of clad wire rods.

## 1. Introduction

High-strength steel wires used in bridge cables are exposed for long periods to coupled environments involving tensile stress, alternating loads, humidity and heat, salt spray, and crevice-corrosion media. Corrosion damage, fretting wear, and fatigue-crack propagation can reduce the load-bearing capacity of steel wires and compromise the service safety of long-span bridge cable systems. Guo and Zhang quantitatively analyzed the coupled effect of corrosion and fatigue on the service life of steel cables and showed that corrosion damage markedly weakens the reliability of service-life assessment [1]. Liu et al. investigated the fatigue behavior and fracture morphology of corroded high-strength bridge cable wires, demonstrating that corrosion pits and coating damage promote fatigue-crack initiation and growth [2]. In addition, studies based on three-dimensional laser scanning and image recognition have enabled quantitative characterization of the corrosion morphology and corrosion grade of galvanized high-strength steel wires [3], whereas investigations of galvanized coatings under fatigue loading have shown that service stress alters the coating-degradation process [4]. At present, cable wires are mainly protected by external measures such as galvanizing or Zn-Al alloy coatings, grease filling, sheathing systems, and dehumidification. However, coating defects, local wear, and moisture enrichment in crevices may still induce localized corrosion, corrosion fatigue, or stress-corrosion cracking. Therefore, implementing corrosion protection at the wire-rod stage, rather than relying solely on external protection during cable service, represents a promising strategy for enhancing the full-life reliability of bridge-cable steel wires.

The new stainless-steel/carbon-steel clad wire rod investigated in this study is a corrosion-resistant composite wire-rod product designed for the preparation of bridge-cable wires. In this material, the carbon-steel core bears the primary mechanical load, whereas the stainless-steel cladding provides corrosion protection, thereby maintaining high strength and subsequent drawing adaptability while reducing stainless-steel consumption. Jia et al. revealed the influence of texture evolution on the torsional performance of cold-drawn pearlitic steel wires, indicating that severe plastic deformation significantly changes the crystallographic orientation and service performance of wire materials [5]. Li et al. further showed that changes in cementite morphology and stored energy induced by cold drawing affect the corrosion behavior and mechanical properties of steel wires [6]. With respect to stainless-steel/carbon-steel clad materials, Liu et al. studied the deformation mechanism and microstructural evolution of longitudinally corrugated hot-rolled stainless-steel clad plates [7]; Wang et al. clarified the interfacial microstructure, mechanical properties, and bonding mechanism of hot-rolled stainless-steel clad plates under different rolling reduction ratios [8]; Chen et al. showed that reduction ratio and high-temperature conditions affect interfacial bonding and cladding corrosion resistance in stainless-steel/carbon-steel clad plates [9]; and Moon et al. revealed the interfacial damage behavior of carbon-steel/stainless-steel clad plates using a hybrid experimental and finite-element approach [10]. Existing studies on clad rebars have also shown that clean-interface assembly, vacuum hot rolling, substrate selection, cross-sectional evolution during rolling, and welding-induced corrosion all influence composite-interface stability and engineering service reliability [11,12,13,14].

Compared with clad plates and clad rebars, clad wire rods are characterized by smaller diameters, larger curvature, coil delivery, and large accumulated deformation during subsequent multi-pass cold drawing. These characteristics impose more stringent requirements on cladding continuity, thickness uniformity, and interfacial stability. However, in cross sections of clad wire rods, the color and grayscale differences between the stainless-steel cladding and the carbon-steel core are limited, the interfacial transition zone is narrow, and specimen preparation may introduce edge reflection, slight cross-sectional ovalization, and local morphological fluctuations. Consequently, conventional manual measurement and threshold-based segmentation methods cannot stably determine cladding boundaries. As this new product moves from laboratory preparation toward batch evaluation, establishing an efficient and stable cross-sectional cladding-recognition method is essential for improving inspection efficiency and enabling quality grading. Nevertheless, current studies on stainless-steel/carbon-steel composite materials have mainly focused on interfacial microstructure, mechanical properties, rolling deformation, corrosion behavior, and service performance of clad plates or clad rebars. Limited attention has been paid to the automatic pixel-level segmentation of the cladding layer in clad wire-rod cross sections. Compared with clad plates and rebars, clad wire rods exhibit a smaller cross-sectional scale, stronger curvature, a thinner annular cladding region, and more pronounced local thickness fluctuations after rolling and drawing. These characteristics make cladding recognition a distinct and more challenging segmentation task rather than a simple extension of existing composite-material characterization or conventional steel-image analysis.

In recent years, deep-learning-based visual inspection methods for steel materials and engineering components have developed rapidly and have been applied to tasks such as rebar size and position recognition, rebar instance segmentation, counting of densely piled steel bars, steel surface-defect segmentation, and wire-rope damage detection [15,16,17,18,19,20,21,22]. Sun et al. applied deep learning to rebar detection and instance segmentation in images, providing an effective approach for target recognition in complex structural components [15]. Huang et al. used synthetic BIM data and domain-adaptation methods to reduce the dependence of on-site rebar instance segmentation on manual annotation [16]. Liu et al. proposed a lightweight convolutional neural network for counting densely piled steel bars, demonstrating the application potential of vision models in batch steel-product quality inspection [17]. For steel surface-defect recognition, U-Net, DeepLabV3+, multi-layer feature-fusion networks, and attention mechanisms have been used for pixel-level defect segmentation and boundary enhancement [18,19,20,21,22]. These studies indicate that deep-learning models can automatically learn target boundaries, texture differences, and local morphological features from complex images and generally exhibit stronger robustness than traditional threshold-based methods under low contrast, nonuniform illumination, and complex backgrounds.

Recent industrial semantic-segmentation studies for metallurgical images and steel defect detection have mainly focused on surface defects, cracks, inclusions, and welding lines using U-Net-based models, DeepLabV3+, attention mechanisms, or feature-fusion networks. Although these methods perform well in defect-region extraction, most are designed for targets with relatively distinguishable texture or grayscale differences from the background. In contrast, this study focuses on thin annular cladding segmentation in cross-sectional images, where the core–cladding contrast is weak, the interfacial transition zone is narrow, and local thickness fluctuations are common. Therefore, ResNet50, ASPP, and CBAM are integrated into a U-Net-based framework to improve semantic feature extraction, multi-scale perception, and boundary refinement for this specific task.

However, directly applying existing semantic segmentation models to clad wire-rod cross-sectional images remains challenging because the cladding layer appears as a thin closed annular region with weak core–cladding contrast, a narrow interfacial transition zone, and local thickness fluctuations. Unlike conventional steel surface-defect segmentation or rebar-recognition tasks, this problem requires more precise preservation of weak boundaries and locally thin cladding regions. Although U-Net can recover spatial details through skip connections, its conventional encoder may be insufficient for extracting discriminative deep features from weak-contrast interfaces, while repeated downsampling may weaken thin-boundary representation. PSPNet and DeepLabV3+ are effective for general semantic segmentation, but they are not specifically designed for attention-guided refinement of ambiguous interfaces and local boundary continuity in clad wire-rod cross sections. To address these limitations, this study proposes an improved U-Net framework using ResNet50 to strengthen deep semantic feature extraction, ASPP to enhance multi-scale contextual modeling, and CBAM to improve attention-guided boundary refinement, thereby providing a high-precision vision-based basis for cladding-thickness measurement, eccentricity calculation, circumferential-uniformity assessment, and batch quality inspection.

## 2. Materials and Methods

The pixel-level recognition performance of deep-learning models depends strongly on dataset size, annotation accuracy, and sample diversity. To enable the model to learn image features of clad wire-rod cross sections under different specimen-preparation states, illumination conditions, color-development effects, and circumferential thickness fluctuations, a cross-sectional image dataset of clad wire rods was constructed. Since no public dataset is currently available for cladding recognition in clad wire rods, original cross-sectional images were acquired using a camera, and training samples were generated through manual fine annotation and data augmentation. This dataset provided the basis for subsequent training and evaluation of the cladding semantic-segmentation model.

### 2.1. Original Data Acquisition

**Figure 1:**
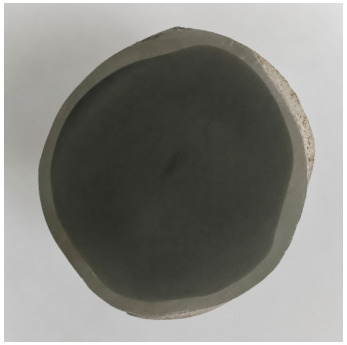
Sample after treatment in a 4% nitric-acid alcohol solution.

To enhance the distinguishability of the cladding region, a standardized cross-sectional image-acquisition procedure was used. Clad wire-rod specimens were cut into approximately 3 cm sections perpendicular to the axial direction, and the target cross sections were ground to obtain flat observation surfaces. The sections were then etched in a 4% nitric-acid alcohol solution for 110 s to slightly corrode the carbon-steel core and improve the color contrast with the stainless-steel cladding, as shown in Figure 1. Each specimen was finally placed on a white background plate, and images were acquired after adjusting the camera axis, focus, and magnification to ensure a clear cross-sectional outline.

**Figure 2:**
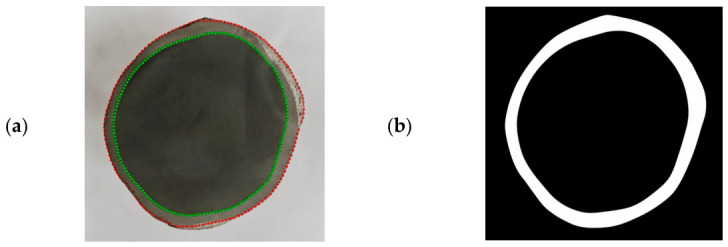
Contour annotation and binary-mask generation: (**a**) contour annotation; (**b**) binary mask image.

A total of 300 cross-sectional images of clad wire rods were collected as the original dataset. The stainless-steel cladding region was manually annotated at the pixel level using Labelme. To improve boundary-annotation accuracy, no fewer than 200 contour control points were assigned to each image, and the point density was increased appropriately in regions with large curvature changes to accurately preserve the cladding-boundary morphology, as shown in Figure 2a. After annotation, each image was divided into cladding and non-cladding classes, and binary mask images in PNG format were generated for subsequent training and evaluation of the semantic-segmentation model, as shown in Figure 2b.

### 2.2. Data Augmentation

**Figure 3:**
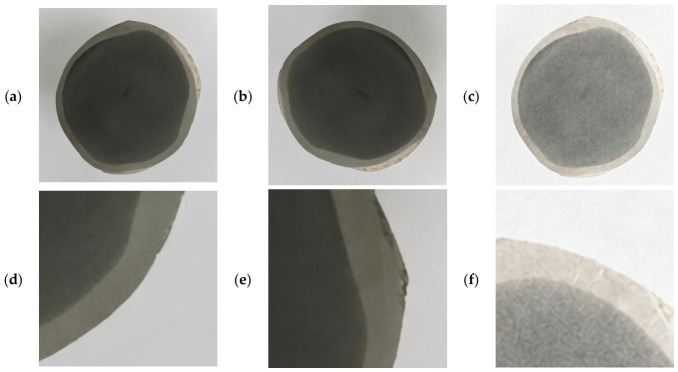
Data-augmentation procedure: (**a**) original image; (**b**) rotation; (**c**) brightness perturbation and Gaussian noise; (**d**) original crop; (**e**) rotated crop; (**f**) brightness/noise crop.

To expand the sample size and improve model robustness to posture variation, illumination fluctuation, and imaging noise, OpenCV was used to augment the 300 original image-mask pairs. First, the images were rotated by 90°, 180°, and 270°. Subsequently, random brightness changes with a factor of 0.7–1.3 and Gaussian noise with a mean range of 1–30 were introduced to simulate imaging disturbances during actual acquisition. Finally, according to the position and size of the cladding region, the augmented images were cropped into 896 × 896-pixel patches for model training and testing. Figure 3a–c show examples of augmentation before cropping, whereas Figure 3d–f show samples in the final dataset.

After initial augmentation by rotation, brightness perturbation, and noise addition, each original image with a resolution of 2700 × 2700 pixels was expanded into eight image groups. Further local cropping according to the position and scale of the cladding region generated 4–8 valid patches per image group, with the exact number depending on the distribution range of the cladding. Each final cropped image-mask pair had a resolution of 896 × 896 pixels. To avoid data leakage, dataset partitioning was performed at the original-image level rather than at the augmented-patch level. Specifically, patches generated from the same original cross-sectional image were assigned only to the same subset and were not allowed to appear simultaneously in the training, validation, and test sets. In total, 18,566 cross-sectional image samples of clad wire rods were constructed. The dataset was first divided into a model-development set and an independent test set at an 8:2 ratio, with 14,853 samples used for model development and 3713 samples used for final testing. Ten percent of the model-development set was further used as the validation set for training monitoring and model selection. Accordingly, 13,368, 1485, and 3713 samples were used for training, validation, and testing, respectively.

### 2.3. U-Net Network Model

U-Net [23] is a representative encoder–decoder fully convolutional neural network, originally proposed by Ronneberger et al. for biomedical image segmentation. By enabling end-to-end pixel-level prediction, U-Net performs semantic image segmentation without relying on fully connected layers, thereby avoiding restrictions on input image size that are commonly associated with conventional convolutional neural networks. Owing to this architectural characteristic, U-Net can achieve high segmentation accuracy and favorable generalization performance even under small-sample or limited-annotation conditions [24]. The network adopts a symmetric U-shaped topology, consisting mainly of a contracting path, an expanding path, and cross-layer skip connections. In the contracting path, successive convolution, nonlinear activation, and pooling operations progressively reduce the spatial resolution of feature maps while increasing the number of feature channels, thereby extracting discriminative contextual semantic information and high-level abstract features. In contrast, the expanding path gradually restores the spatial resolution through upsampling or deconvolution operations and maps deep semantic representations back to the pixel space, ultimately generating segmentation results with a spatial size corresponding to that of the input image.

**Figure 4:**
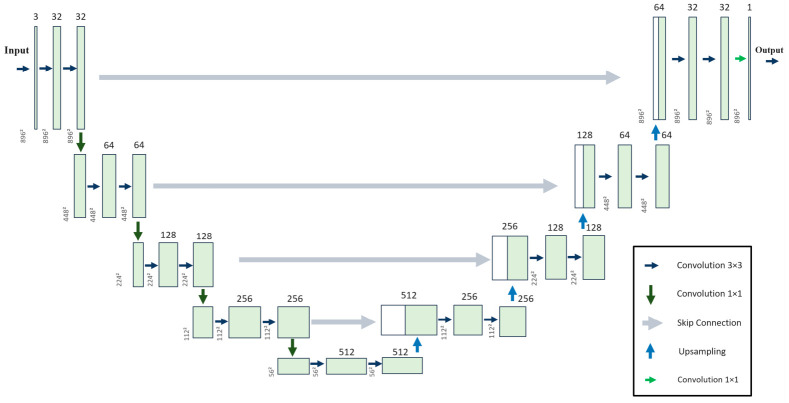
U-Net network architecture.

A key characteristic of U-Net lies in its skip-connection mechanism. By fusing high-resolution shallow features from the encoder with deep semantic features from the corresponding decoder layers, this mechanism preserves global semantic representation while effectively compensating for the loss of boundary, texture, and local detail information caused by downsampling. For cross-sectional images of stainless-steel/carbon-steel clad wire rods, the interface between the cladding and core is typically characterized by a narrow transition zone, low grayscale or color contrast, pronounced local fluctuations in cladding thickness, and complex circumferential boundary morphology. These characteristics impose stringent requirements on the boundary discrimination and detail preservation capabilities of segmentation models. Through hierarchical feature extraction and the fusion of shallow and deep features, U-Net can effectively capture narrow interfaces, weak-contrast boundaries, and subtle local structural features, thereby providing a robust network foundation for accurate cladding-region identification. Therefore, adopting U-Net as the baseline model for cross-sectional image segmentation of clad wire rods can provide reliable pixel-level segmentation results for subsequent cladding-thickness measurement, eccentricity calculation, circumferential-uniformity evaluation, and batch quality inspection. The architecture of the U-Net network is shown in Figure 4.

### 2.4. Design of the Improved U-Net Network

To address the low contrast between the cladding and core regions, the narrow interfacial transition zone, blurred boundary contours, and pronounced local fluctuations in cladding thickness in cross-sectional images of stainless-steel/carbon-steel clad wire rods, this study improves the classical U-Net encoder–decoder framework to enhance pixel-level recognition accuracy and boundary segmentation stability of the cladding region. First, ResNet50 is adopted as the encoder backbone to replace the stacked convolutional feature-extraction structure in the original U-Net. Through residual connections, ResNet50 enables cross-layer feature propagation, effectively alleviating gradient vanishing and network degradation during deep network training, while enhancing the representation capability of the model for complex textures, weak-contrast interfaces, and high-level semantic information [25]. In addition, to improve the compatibility between the encoder output features and the decoder feature-reconstruction process, the convolutional structure of the decoder is moderately simplified. This design reduces redundant parameters and computational complexity while maintaining progressive spatial-resolution recovery and effective fusion of shallow and deep features, thereby lowering the risk of overfitting and improving model generalization.

**Figure 5:**
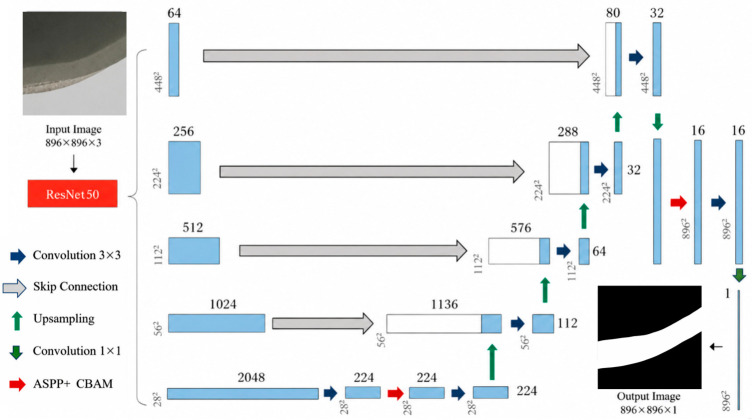
Architecture of the improved U-Net network.

During the feature-fusion stage, the convolutional block attention module (CBAM) and atrous spatial pyramid pooling (ASPP) module are further introduced to enhance the model’s perception of critical boundary regions and multi-scale contextual information. Specifically, CBAM jointly models channel and spatial attention to adaptively strengthen effective feature responses associated with cladding boundaries, locally thin cladding regions, and weak-contrast interfaces, while suppressing background noise and irrelevant texture interference [26]. The ASPP module employs parallel atrous convolutions with different dilation rates to extract multi-scale features and enlarge the receptive field without reducing the spatial resolution of feature maps, thereby improving the model’s adaptability to variations in cladding thickness, complex circumferential boundaries, and local morphological changes [27]. Overall, through the synergistic integration of residual feature extraction, lightweight decoder reconstruction, attention enhancement, and multi-scale contextual modeling, the improved U-Net can more effectively identify weak boundaries and fine details in clad wire-rod cross sections, providing a reliable segmentation basis for subsequent cladding-thickness measurement, eccentricity calculation, and circumferential-uniformity evaluation. The architecture of the improved U-Net is shown in Figure 5.

**Figure 6:**
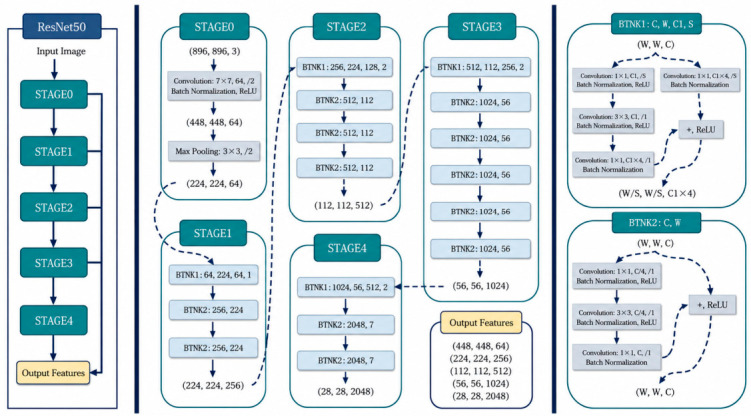
ResNet network architecture.

The backbone network determines the feature-representation capability of a semantic-segmentation model. The depth, scale, and discriminative ability of the extracted features directly affect subsequent decoding and pixel-classification accuracy. Conventional VGG networks are mainly composed of stacked 3 × 3 convolutions and 2 × 2 max-pooling layers. Although this structure is simple and the feature-extraction process is clear, increasing network depth can lead to gradient attenuation, parameter redundancy, and insufficient high-level feature representation [28]. By contrast, ResNet transmits shallow inputs directly to deeper outputs through residual connections, effectively alleviating degradation during deep-network training and enabling the model to extract more discriminative high-level semantic features. Therefore, ResNet50 was selected as the encoder backbone of the improved U-Net to enhance recognition of low-contrast interfaces and complex local boundaries in cross sections of clad wire rods. Its structure is shown in Figure 6.

ResNet50 consists of five successive feature-extraction stages that progressively compress the spatial size of feature maps and increase the channel dimension, thereby obtaining hierarchical features at different scales. To effectively fuse ResNet50 output features with the U-Net decoder, a 3 × 3 convolution was introduced after each skip connection and after the output features of the fifth stage to adjust the number of channels and improve feature compatibility. Meanwhile, the convolutional unit following each skip connection was simplified from two layers to one layer. This design reduces the number of model parameters, lowers the overfitting risk, and improves training efficiency while maintaining feature-fusion capability. In addition, an upsampling operation was added at the end of the network so that the output segmentation map has the same size as the input image, satisfying the requirement for pixel-level cladding recognition.

**Figure 7:**
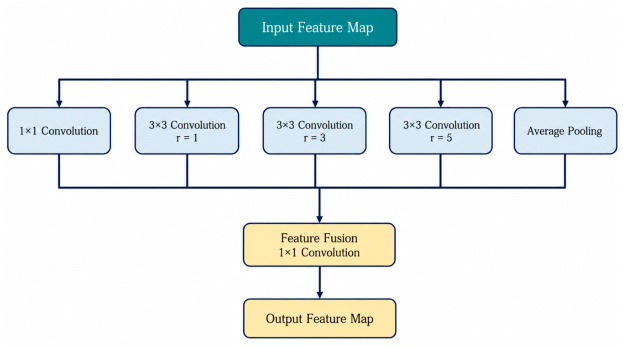
ASPP module architecture.

To further improve adaptability to cladding-edge details and scale variations, ASPP modules were introduced after the deep-feature output of ResNet50 and after the final upsampling operation. The structure of the ASPP module is shown in Figure 7. The ASPP module consists of a 1 × 1 convolution branch, several atrous-convolution branches with different dilation rates, and a global-average-pooling branch. It can enlarge the receptive field without significantly reducing feature-map resolution and can fuse local details with global contextual information. This structure helps the model capture narrow interfaces, locally thin cladding regions, and boundary morphologies at different scales in clad wire-rod cross sections.

**Figure 8:**
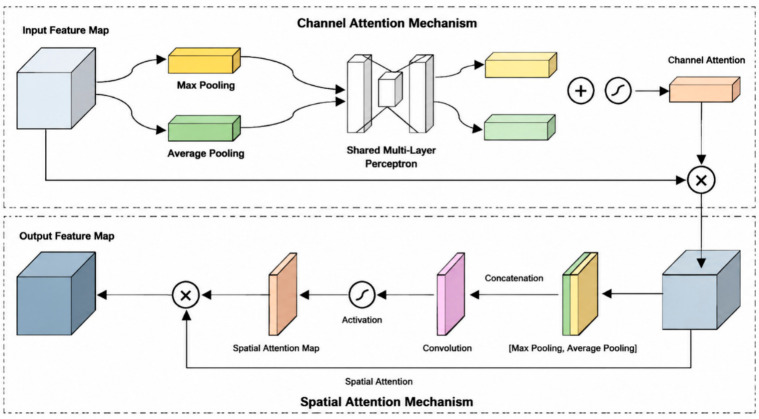
CBAM module architecture.

After the ASPP module, a CBAM attention module was embedded to enhance the feature response to critical cladding regions. Its structure is shown in Figure 8. CBAM is composed of channel-attention and spatial-attention submodules arranged sequentially. Channel attention models the importance of different feature channels through global average pooling and max pooling, thereby highlighting effective semantic information related to cladding recognition. Spatial attention assigns weights along the spatial dimension, enabling the model to focus more strongly on interfacial boundaries, locally weak regions, and areas prone to missegmentation. Through the synergistic effect of ResNet50, ASPP, and CBAM, the improved U-Net can more fully extract deep semantics, multi-scale context, and local boundary details, thereby improving the segmentation accuracy and stability of the cladding region in clad wire-rod cross sections.

## 3. Experiments and Results

### 3.1. Experimental Environment and Parameter Setting

The experiments were performed on a desktop computer running Windows 10. The main hardware configuration included an NVIDIA GeForce RTX5070 GPU with 8 GB of memory, an Intel(R) Core(TM) Ultra 9 processor, and 32 GB of RAM. The software environment consisted of Python 3.6, Keras 2.6.0, TensorFlow 2.6, and OpenCV 3.4.2.16. To ensure fair comparison, all models were trained, tested, and evaluated under the same hardware and software environment.

Model training and validation were conducted using the self-built dataset. The Adam optimizer was used with a batch size of two, an initial learning rate of 0.0001, 20 training epochs, and cross-entropy loss. The model with the highest validation mIoU was saved for final testing, and each model was independently trained three times using fixed dataset-splitting rules and different random seeds. For the proposed model, with an input size of 896 × 896 pixels, the peak GPU memory consumption was approximately 7.1 GB, one complete training run required approximately 5.8 h, the average inference time was approximately 52 ms per image, and the computational complexity was approximately 186.4 GFLOPs. These results indicate that although the proposed model is larger than the baseline U-Net, it can still be trained and tested on a single 8 GB GPU for offline cladding-quality inspection.

### 3.2. Ablation Experiments on the Improved U-Net Network

**Figure 9:**
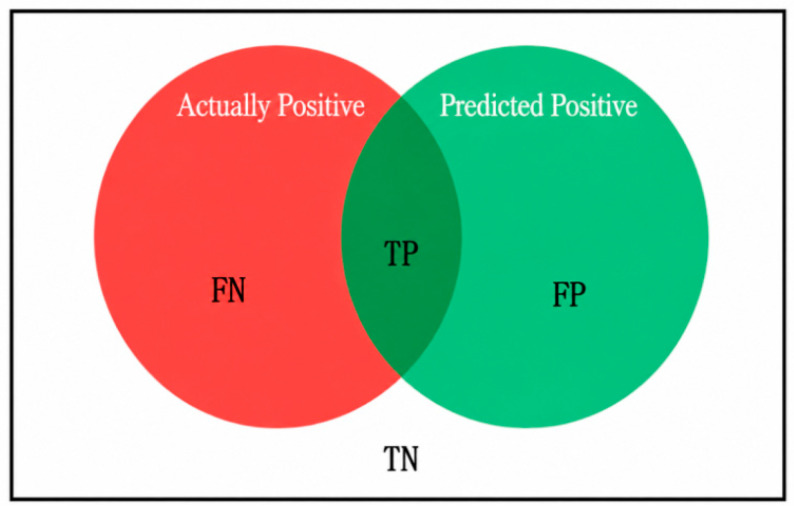
Binary classification relationship between ground truth and prediction.

To verify the effectiveness of the proposed local cladding-recognition model, ablation experiments were conducted using the improved U-Net as the research object. The number of model parameters, mean pixel accuracy (MPA), intersection over union (IoU), and mean intersection over union (mIoU) were selected as the main quantitative performance-evaluation metrics. Among them, MPA was used to evaluate pixel-level classification accuracy, whereas IoU and mIoU were used to evaluate the regional overlap between the predicted cladding mask and the ground-truth annotation. Considering that cladding-boundary continuity is important for subsequent thickness measurement and uniformity assessment, boundary-level differences were further analyzed and are discussed in detail in the subsequent visual comparison and enlarged boundary-view analysis. For the binary classification problem in cladding recognition, each pixel can be classified into one of four categories according to the correspondence between the pixel-level ground-truth label and the model prediction: true positive (TP), false positive (FP), true negative (TN), and false negative (FN), as shown in Figure 9. By counting the numbers of these four pixel types, a confusion matrix for model-performance analysis can be constructed, as shown in Table 1.

Mean pixel accuracy (MPA) measures the average recognition ability of a model across all pixel classes. It is calculated by first determining the proportion of correctly classified pixels in each class and then averaging the pixel-recognition accuracies over all classes. Based on the confusion matrix, MPA can be calculated using Equation (1).

MPA = 1/2 [TP/(TP + FN) + TN/(TN + FP)](1)

Intersection over union (IoU) evaluates the segmentation accuracy for a specific pixel class and is defined as the ratio of the intersection to the union between the predicted result and the ground-truth label for that class. For the cladding class, IoU can be calculated using Equation (2).

IoU = TP/(TP + FP + FN)(2)

Mean intersection over union (mIoU) is obtained by averaging the IoU values over all classes and provides an overall evaluation of segmentation performance. Based on the confusion matrix, mIoU can be calculated using Equation (3).

mIoU = 1/2 [TP/(TP + FP + FN) + TN/(TN + FP + FN)](3)

The experimental results for different model configurations are shown in Table 2. The values are reported as mean ± standard deviation over three independent runs with different random seeds. Experiment 1 corresponds to the original U-Net, whose backbone adopts a VGG-based encoder–decoder structure with an initial channel number of 16 and a network depth of four.

In Experiment 2, the encoder backbone of the original U-Net was replaced with a ResNet architecture, while the number of convolutional layers in the decoder was correspondingly reduced. Although this modification led to a substantial increase in model parameters compared with the original U-Net, all evaluation metrics, including MPA, IoU, and MIoU, were markedly improved. This indicates that the ResNet backbone can enhance deep feature propagation and semantic representation through residual connections, effectively mitigating gradient vanishing and feature degradation that may occur as network depth increases. Consequently, the model’s ability to recognize cladding regions, weak-contrast interfaces, and complex boundary features was improved. In Experiment three, a CBAM attention module was further incorporated into the ResNet-based encoder. With only a slight increase in the number of parameters, both IoU and MIoU were improved, demonstrating that the joint modeling of channel and spatial attention can guide the model to focus more effectively on cladding boundaries, locally thin cladding regions, and fine structural details, while suppressing background noise and irrelevant texture interference. This contributes to improved accuracy and stability in cladding segmentation.

In Experiment four, the ASPP module was introduced on the basis of the ResNet network. Although this further increased the model parameter count, the segmentation metrics showed notable improvement, indicating that atrous spatial pyramid pooling can extract multi-scale contextual information through parallel atrous convolutions with different dilation rates. This enhances the model’s adaptability to variations in cladding thickness, complex circumferential contours, and local morphological changes. In Experiment five, CBAM and ASPP were introduced simultaneously, enabling a more favorable balance between model complexity and segmentation performance. Compared with Experiment two, the number of parameters increased by only 2.9 M, whereas MPA, IoU, and MIoU increased by 0.71, 3.71, and 2.09 percentage points, respectively. These results suggest that the attention mechanism and multi-scale contextual modeling exhibit strong complementarity and can jointly improve the representation of critical cladding regions and weak boundary features. Compared with the original U-Net, the final improved model increased MPA, IoU, and MIoU by 1.38, 9.19, and 5.17 percentage points, respectively, verifying the effectiveness of ResNet-based feature extraction, CBAM-based attention enhancement, and ASPP-based multi-scale feature fusion for cross-sectional image segmentation of clad wire rods. Overall, these results indicate that the improved model can enhance cladding-region recognition accuracy, boundary continuity, and segmentation stability, thereby providing more reliable pixel-level segmentation results for subsequent cladding-thickness measurement and circumferential-uniformity evaluation.

### 3.3. Comparison of Recognition Effects Among Models

To further evaluate the comparative performance of the improved U-Net model in cross-sectional cladding segmentation, several representative semantic segmentation networks were selected for comparison. Under the same experimental environment and training strategy, representative semantic segmentation models, including the original U-Net, PSPNet [29], and DeepLabV3+ [30], were constructed and trained with different backbone feature-extraction networks, such as VGG, MobileNetV2 [31], and ResNet. All models were trained and tested using the same cross-sectional image dataset of clad wire rods, with identical data partitioning, input size, and evaluation metrics, thereby minimizing the influence of experimental-condition differences on model performance comparison and ensuring the fairness and reproducibility of the comparative results.

**Figure 10:**
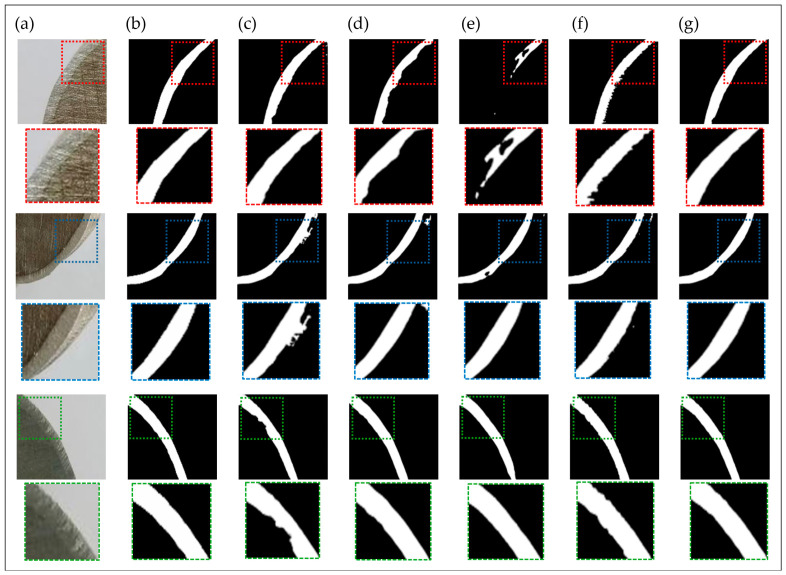
Visual comparison of segmentation results obtained with different backbone/network configurations: (**a**) original image; (**b**) ground truth; (**c**) U-Net/VGG; (**d**) PSPNet/MobileNetV2; (**e**) PSPNet/ResNet; (**f**) DeepLabV3+/MobileNetV2; and (**g**) the proposed model. The marked regions are enlarged to highlight boundary-segmentation details.

By introducing different types of semantic segmentation networks and backbone architectures, the models can be comprehensively evaluated in terms of feature-extraction capability, multi-scale contextual modeling, boundary-detail recovery, and overall segmentation stability. Specifically, PSPNet primarily relies on a pyramid pooling structure to capture global contextual information, whereas DeepLabV3+ enhances multi-scale feature representation through atrous convolution and an encoder–decoder architecture. Meanwhile, different backbone networks exhibit distinct characteristics in feature abstraction capability and model complexity. Although Transformer-based segmentation architectures have shown strong global-context modeling capability in many vision tasks, they were not included as the main comparison models in this study because the present work focuses on a task-oriented improvement of U-Net under limited original cross-sectional images and single-GPU computational conditions. In addition, Transformer-based models generally require larger-scale training data and higher computational cost to fully exploit self-attention mechanisms. Therefore, this study mainly compared the proposed method with representative CNN-based segmentation models commonly used in industrial image analysis. Comparing these models with the proposed improved U-Net enables a more comprehensive assessment of the practical advantages of the proposed method in recognizing weak-contrast interfaces, narrow cladding regions, and complex circumferential boundaries. The recognition results of different models on cross-sectional images of clad wire rods are shown in Figure 10.

The enlarged boundary views in Figure 10 further demonstrate that the proposed model preserves more continuous cladding boundaries and produces fewer local omissions than the comparison models, particularly in weak-contrast interfaces and locally thin cladding regions. This visual improvement is consistent with the higher IoU and mIoU values reported in Table 3. Compared with PSPNet, which shows obvious missed detections and boundary misclassifications, U-Net-VGG and DeepLabV3+ achieve better segmentation results but still suffer from discontinuous contours and incomplete recognition of fine details. In contrast, the proposed improved U-Net shows higher consistency with manual annotations in both overall morphology and local boundary details. These results indicate that the integration of the ResNet backbone, CBAM attention mechanism, and ASPP module effectively enhances the representation of weak-boundary, thin-region, and complex-contour features, thereby improving the accuracy and robustness of local cladding segmentation.

To further quantitatively evaluate the performance differences among different semantic segmentation models in the recognition of cross-sectional images of clad wire rods, this study selected the number of model parameters, mean pixel accuracy (MPA), intersection over union (IoU), and mean intersection over union (MIoU) as the main evaluation metrics, thereby enabling a comprehensive comparison of segmentation accuracy, regional overlap, and model complexity. Specifically, the number of model parameters reflects the structural complexity of the network and its computational resource requirements. MPA measures the average pixel-level prediction accuracy across different categories and thus indicates the overall classification capability of the model for different regions. IoU primarily evaluates the degree of overlap between the predicted cladding region and the manually annotated region, serving as a key indicator for assessing cladding recognition accuracy. MIoU, calculated as the average IoU across all categories, provides an integrated measure of the stability and generalization performance of the model in the overall semantic segmentation task. Through the joint analysis of these quantitative metrics and the enlarged boundary views in Figure 10, the differences among various networks in terms of recognition accuracy, regional integrity, boundary continuity, and model complexity can be more comprehensively compared. The quantitative evaluation results of different models are presented in Table 3. The values are reported as mean ± standard deviation over three independent runs with different random seeds.

The results reveal clear differences among the compared segmentation networks in both complexity and accuracy. PSPNet-MobileNetV2 is lightweight but shows limited cladding-recognition accuracy, indicating insufficient extraction of weak-contrast boundaries and subtle local features. Using ResNet as the PSPNet backbone improves feature extraction but yields only marginal metric gains, suggesting that pyramid pooling remains inadequate for narrow cladding regions. DeepLabV3+ achieves the lowest overall accuracy, likely because its atrous-convolution-based multi-scale representation cannot fully preserve continuous cladding details under low-contrast and complex boundary conditions.

In contrast, although the original U-Net has the fewest parameters and offers a simple architecture with relatively high training efficiency, its basic convolutional encoder limits its capacity to represent deep semantic information and complex boundary features. After incorporating the ResNet backbone, CBAM attention mechanism, and ASPP multi-scale contextual module, the recognition performance of U-Net was improved, with MPA, IoU, and mIoU higher than those of the comparison models under the same experimental conditions. These results indicate that the improved U-Net can better represent weak-contrast interfaces, locally thin cladding regions, and complex boundary details. Consequently, it provides improved segmentation accuracy and boundary stability for local cladding recognition, although the increased model complexity should also be considered in practical deployment.

### 3.4. Analysis of Missegmentation Cases

Although the proposed improved U-Net achieved the best overall segmentation performance, several missegmentation cases were still observed in challenging local regions. The main errors occurred near weak-contrast interfaces where the color difference between the stainless-steel cladding and the carbon-steel core was insufficient, causing slight boundary deviations between the predicted mask and the ground-truth annotation. Local omissions were also occasionally observed in extremely thin cladding regions, especially when the interfacial transition zone was narrow or blurred after chemical color development. In addition, uneven etching, edge reflection, and local background interference could lead to small false-positive responses near the outer boundary of the specimen.

Compared with U-Net, PSPNet, and DeepLabV3+, the proposed model reduced most of these errors by combining deep semantic extraction, multi-scale contextual perception, and attention-guided boundary refinement. In particular, ASPP improved the recognition of cladding regions with variable thickness, while CBAM helped the model focus on weak boundaries and locally thin regions. Nevertheless, complete elimination of missegmentation remains difficult because part of the boundary ambiguity originates from specimen preparation, chemical etching, and imaging conditions rather than the network structure alone. Future work will further improve image acquisition consistency, incorporate more difficult boundary samples, and introduce boundary-specific quantitative metrics, such as boundary F-score and Hausdorff distance, to further evaluate cladding-boundary localization accuracy.

## 4. Conclusions

This study proposed an improved U-Net cladding-segmentation method integrating ResNet50, ASPP, and CBAM to address the low interfacial contrast, narrow boundary transition zone, pronounced local thickness fluctuation, and insufficient consistency of manual measurements encountered in cross-sectional quality inspection of stainless-steel/carbon-steel clad wire rods for bridge cables. The main conclusions are as follows:

(1) A standardized workflow for cross-sectional recognition of stainless-steel/carbon-steel clad wire rods was established, and a dataset containing 18,566 annotated samples was constructed. The dataset covers variations in morphology, illumination, specimen posture, and local cladding thickness, providing a reliable basis for training and evaluating intelligent cladding-recognition models.
(2) By introducing ResNet50 into the U-Net encoder and combining ASPP-based multi-scale contextual modeling with CBAM-based attention enhancement, the proposed model effectively improved the representation of low-contrast interfaces, locally thin cladding regions, and complex circumferential boundaries. Test results showed that the improved U-Net achieved an MPA, cladding IoU, and mIoU of 97.29%, 88.82%, and 93.72%, respectively, representing increases of 1.38, 9.19, and 5.17 percentage points compared with the original U-Net.
(3) The ablation experiments and multi-model comparison demonstrate that ResNet50, ASPP, and CBAM all contribute positively to model-performance improvement. ASPP enhances multi-scale boundary-feature extraction, whereas CBAM increases the model attention to key cladding regions and locations prone to missegmentation. Compared with representative semantic-segmentation models such as U-Net, PSPNet, and DeepLabV3+, the proposed model achieved better recognition accuracy, boundary continuity, and local-detail preservation. It therefore provides a high-precision image-recognition basis for cladding-thickness measurement, eccentricity calculation, circumferential-uniformity assessment, and batch quality inspection. These results indicate that the proposed method is an effective task-oriented improvement for this specific industrial segmentation problem, although it is not intended as a fundamentally new general-purpose segmentation methodology.

## Figures and Tables

**Table 1:** Confusion matrix for binary classification.

Ground Truth	Prediction	
	Positive (Cladding)	Negative (Background)
Positive (cladding)	TP	FN
Negative (background)	FP	TN

**Table 2:** Ablation experiments for the improved U-Net.

Experiment	Input Size	Backbone	Parameters	MPA (%)	IoU (%)	mIoU (%)
1	896	VGG	1.9 M	95.91 ± 0.08	79.63 ± 0.32	88.55 ± 0.18
2	896	ResNet	31.8 M	96.58 ± 0.07	85.11 ± 0.27	91.63 ± 0.15
3	896	ResNet + CBAM	32.4 M	96.83 ± 0.06	86.54 ± 0.24	92.41 ± 0.13
4	896	ResNet + ASPP	34.6 M	97.12 ± 0.05	88.06 ± 0.21	93.38 ± 0.11
5	896	ResNet + ASPP + CBAM	34.7 M	97.29 ± 0.05	88.82 ± 0.18	93.72 ± 0.10

**Table 3:** Performance comparison among different semantic-segmentation models.

Model	Input Size	Backbone	Parameters	MPA (%)	IoU (%)	mIoU (%)
U-Net	896	VGG	1.9 M	95.91 ± 0.08	79.63 ± 0.32	88.55 ± 0.18
U-Net	896	ResNet	31.8 M	96.58 ± 0.07	85.11 ± 0.27	91.63 ± 0.15
PSPNet	896	MobileNet V2	2.4 M	95.29 ± 0.10	78.11 ± 0.40	87.68 ± 0.23
PSPNet	896	ResNet	14.4 M	95.22 ± 0.09	79.63 ± 0.36	88.53 ± 0.21
DeepLabV3+	896	MobileNet V2	2.7 M	94.54 ± 0.11	77.57 ± 0.43	87.35 ± 0.25
Improved U-Net	896	ResNet + ASPP + CBAM	34.7M	97.29 ± 0.05	88.82 ± 0.18	93.72 ± 0.10

## Data Availability

The original contributions presented in this study are included in the article. Further inquiries can be directed to the corresponding author.
